# Association of adipokines and adhesion molecules with indicators of obesity in women undergoing mammography screening

**DOI:** 10.1186/1743-7075-9-97

**Published:** 2012-10-31

**Authors:** Caroline Isoppo de Souza, Daniela Dornelles Rosa, Betina Ettrich, Gabriela Hermann Cibeira, Juliana Giacomazzi, Paloma Tusset, Patrícia Ashton-Prolla, Lidia Rosi Medeiros, Maira Caleffi, Eurico Camargo Neto, Emilio Hideyuki Moriguchi, Marcia Silveira Graudenz

**Affiliations:** 1 Programa de Pós Graduação em Medicina: Ciências Médicas, Universidade Federal do Rio Grande do Sul (UFRGS), Rio Grande do Sul, Brasil; 2 Hospital Moinhos de Vento, Porto Alegre, Rio Grande do Sul, Brasil; 3 Grupo Brasileiro de Estudos do Câncer de Mama (GBECAM), São Paulo, Brasil; 4 Programa de Pós Graduação em Cardiologia e Ciências Cardiovasculares, Universidade Federal do Rio Grande do Sul (UFRGS), Rio Grande do Sul, Brasil; 5 Laboratório de Medicina Genômica do Hospital de Clínicas de Porto Alegre (HCPA), Rio Grande do Sul, Brasil; 6 Instituto Nacional de Genética Médica Populacional (INAGEMP), Porto Alegre, Brasil; 7 Laboratório Nobel Sociedade Simples Ltda, Rio Grande do Sul, Brasil; 8 Núcleo Mama Porto Alegre (NMPOA), Rio Grande do Sul, Brasil; 9 Departamento de Patologia, Universidade Federal do Rio Grande do Sul, Rio Grande do Sul, Brasil; 10 Departamento de Genética, Universidade Federal do Rio Grande do Sul, Rio Grande do Sul, Brasil; 11 Hospital de Clinicas de Porto Alegre (HCPA), , Rio Grande do Sul, Brazil

**Keywords:** Obesity, Adiponectin, Plasminogen Activator Inhibitor type-1, Vascular Cell Adhesion Molecule-1, Intercellular Adhesion Molecule-1

## Abstract

**Background:**

The soluble cell adhesion molecules and adipokines are elevated in patients with obesity, hypertension, type 2 diabetes mellitus, breast cancer and atherosclerosis**.**

**Objective:**

To investigate the relationship between anthropometric profile, dietary intake, lipid profile and fasting glycemia with serum levels of adipokines (adiponectin and PAI-1) and adhesion molecules (ICAM-1 and VCAM-1) in women without breast cancer undergoing routine mammographic screening.

**Design:**

Transversal study.

**Subjects:**

One hundred and forty-five women over 40-years old participated in this study.

**Results:**

In 39.3% of cases the BMI was above 30 kg/m^2^; 46.9% had hypertension, 14.5% had type 2 Diabetes Mellitus, 31.7% had dyslipidemia and 88.3% presented a waist-to-hip ratio ≥ 0.8. A linear correlation was found between serum levels of PAI-1 and triglycerides, between serum levels of PAI-1 and WHR and between serum levels of VCAM-1 and BMI.

**Conclusion:**

We found a high prevalence of obesity and metabolic syndrome. PAI-1 and VCAM-1 levels were correlated with clinical indicators of obesity and overweight.

## Background

Forty-nine percent of the Brazilian adult population is overweight and 14.8% is obese [1]. The southern region of the country concentrates the highest percentage of overweight and obesity among women (51.6% and 19.6%, respectively) [2,3]. Obesity has been identified as a risk factor for breast carcinoma in postmenopausal women and is associated with worse prognosis [4]. Adipose tissue is an important source of peripheral conversion of estrogen via the aromatase enzyme, which may induce mitogenic activity on mammary epithelium. The association between obesity and insulin resistance is well established and there is evidence that insulin and insulin-like growth factors are involved in the development of breast cancer [4].

Obesity alters the expression of adipokines , which are cytokines secreted by the adipose tissue [5,6]. The adipokine adiponectin (acrp30, adipoQ, apM1 gene product) seems to exert a potent anti-inflammatory and vascular protective effect. Reduced levels of adiponectin have been implicated in the pathogenesis of obesity, type 2 diabetes mellitus and breast cancer in postmenopausal women [6-10].

Plasminogen activator inhibitor type-1 (PAI-1) is another adipokine that has been used as biomarker of the interactions between the fibrinolytic system, inflammation, oxidative processes, adipose tissue, metabolic syndrome and atherosclerotic diseases [11,12]. It has also a relevant role in tumoral adhesion, cellular migration and angiogenesis. Elevated levels of PAI-1 seem to be associated with worse prognosis in breast cancer [13].

The soluble cell adhesion molecules are elevated in patients with obesity, hypertension, type 2 diabetes mellitus and atherosclerosis [14-16]. Elevated levels of the intercellular adhesion molecule-1 (ICAM-1) and vascular cell adhesion molecule-1 (VCAM-1) are found in stage IV breast cancer patients and are associated with worse prognosis [17].

This study aimed to investigate the relationship between anthropometric profile (body mass index and waist-to-hip ratio), food intake frequency, lipid profile and fasting glycemia with serum levels of adipokines (adiponectin and PAI-1) and adhesion molecules (ICAM-1 and VCAM-1) in a subgroup of women derived from a cohort of 10,000 women undergoing annual mammographic screening in Southern Brazil (Núcleo Mama Porto Alegre - NMPOA). The NMPOA is a public-private research project funded by the Associação Hospitalar Moinhos de Vento in partnership with the Porto Alegre City Health Department, which aims to reduce by 20% the incidence of breast cancer in the city. Details of the NMPOA project have been described elsewhere [18].

## Methods

### Patients

A consecutive and unselected sample of 145 women without breast cancer (age over 40 years) enrolled in a mammography screening program in the city of Porto Alegre (*Núcleo Mama Porto Alegre* – NMPOA cohort) was recruited for this study during routine mammographic visits between January and April 2009. Study approval was obtained from the ethics committees of the participating institutions (Hospital Moinhos de Vento number 2009/44 and Hospital de Clínicas de Porto Alegre number 08–649) and all individuals recruited for the study signed an informed consent form. Demographic and clinical information as well as results from mammographic screening were obtained from chart review.

### Blood collection and analysis of adipokines, PAI-1, ICAM-1 and VCAM-1

Peripheral blood samples were obtained after a 12h fasting period by venipuncture of the antecubital vein. All samples were centrifuged at 3000 rpm for 10 minutes, and serum was aliquoted and stored at - 80°C. Serum levels of adipokines (Apn and PAI-1) and adhesion molecules (ICAM-1 and VCAM-1) were analyzed by the multiplex immunoassay method in an automatic equipment Luminex® 200™ IS (Luminex Corporation, Austin, Texas, USA), using the commercial kit ‘Human cardiovascular disease (CDV) panel 1 LINCOplex’ (LINCO Research, EUA) for simultaneous measurements of Apn, PAI-1, ICAM-1, VCAM-1. For the analysis, samples were thawed, centrifuged at 3000 rpm for 10min and diluted in the appropriate sample matrix in 1:400, according to the kit instructions. The intra- and inter-analysis variations of the biomarkers were 9.2% and 15.9% for adiponectin, 11.8% and 12.5% for PAI-1, 7.9% and 9.7% for ICAM-1 and 4.5% and 8.5% for VCAM-1, respectively.

### Anthropometric measures and dietary intake

Anthropometric measures (weight, height, waist circumference and hip circumference) were measured according to the recommendations of the Brazilian Ministry of Health [19,20]. Body mass index (BMI) was calculated by dividing weight in kilograms (kg) by height in square meters (m^2^) and categorized according to the World Health Organization (WHO) criteria [20]. The waist-to-hip ratio (WHR) was established by dividing the value of the waist circumference by the hip circumference. For women, values ≥0.8 are accepted as a risk factor for complications related to obesity [19]. Body weight was measured on a digital platform scale with a capacity of 150kg. Height was measured with a 2m stadiometer attached to the scale, with patients barefoot and wearing as little clothing as possible. Circumferences were measured with a standard inelastic and flexible tape. The entire NMPOA cohort study was described in 2009 [2].

The dietary pattern of the participants was characterized by a food frequency questionnaire (FFQ) applied at the time of blood collection. The FFQ consisted of 103 items designed to ascertain in detail the quantities and kinds of foods consumed over the previous year [1]. Study subjects were asked to report their frequency of consumption (by day, week, month, or year) and portion size of each food item consumed, over the one-year period preceding confirmation of disease. The food groups included in the FFQ were vegetables (carrots, onion, lettuce, tomatoes), potatoes, fruit, cereals (categories of bread, pasta, rice, and pizza), meat and meat products, fish, dairy products (categories of cheese, milk, and yogurt), eggs, cakes, linseed, beans, added fat, added sugar and alcoholic beverages. Data on dietary intake were evaluated with the *DietWin*® software and classified according to the Brazilian Table of Nutrition (BTN) [1].

### Statistical analysis

A descriptive analysis of all variables was performed for the quantitative variables, using the mean and standard deviation. The categorical clinical variables were expressed as proportions. Student’s t-test was used to compare means. Pearson’s correlation coefficient was used to measure the degree of association between two numerical variables. When there was a linear trend, means were compared using the ANOVA test. We used multivariate analysis to control for confounding variables, using p <0.1 for selecting covariates for the model. The statistical analysis was performed using SPSS version 17.0 (Statistical Package for Social Science, SPSS Inc, Illinois, EUA). The significance level adopted was p<0.05.

## Results

### Population sample

The overall clinical, anthropometric and dietary characteristics of the sample are described in Table 1.

**Table 1:** Characteristics of the sample (n=145)

**Variables**	**Values (± SD)**
Mean age (years)	55.74 (±8,01)
Mean BMI (kg/m^2^**)**	29.46 (±5,6)
Mean WHR	0.88 (±0,06)
WHR ≥ 0.8	88.3%
Race (%)	
White	76.6
Black	23.4
Mean age of menopause (years)	46 (±5.6)
Postmenopausal status (%)	64.5
Mean number of meals/day	3.94 (±1)
Mean energy intake (kcal/day)	2107 (±599)
Mean fasting glycemia levels (mg/dl)	105.38 (±26.9)
Mean triglycerides levels (mg/dl)	165.63 (±99)
Mean total cholesterol levels (mg/dl)	205 (±44)
Mean HDL cholesterol levels (mg/dl)	53.48 (±15.5)
Mean LDL cholesterol levels (mg/dl)	115.7 (±40.5)
Prevalence of diabetes (%)	14.5
Prevalence of hypertension (%)	46.9
Prevalence of dyslipidemia (%)	31.7
Use of oral hypoglycemic agents (%)	6.9
Use of statins (%)	12.4
Physical activity (%)*	35.2
BMI (kg/m^2^)	
<18.5	0.7
18.5-24.9	22.8
25-29.9	37.2
30-34	24.8
35-39.9	9
≥40	5.5

*BMI – body mass index;* WHR - waist-hip ratio; HDL-High Density Lipoprotein; LDL-Low Density Lipoprotein.

* Defined as a minimum of 30 minutes of exercises 2 times a week.

### Serum biomarkers

Serum levels of Adiponectin, PAI-1, VCAM-1 and ICAM-1 in the sample are described in Table 2.

**Table 2:** Values of Adiponectin PAI-1, VCAM-1 and ICAM-1

**Variables (ng/ml)**	**Mean values (±SD)**
Adiponectin	279.4 (±61.3)
PAI-1	90.31 (±37.9)
ICAM −1	56.9 (±39.2)
VCAM-1	321 (±60.8)

### Association of dietary intake and correlation between energy and serum levels of adipokines and adhesion molecules

Food consumption was dichotomized by number of meals (up to 4 and more than 4) (Table 3). No association was found between dietary intake and amount of calories/day ingested with serum levels of adipokines and adhesion molecules.

**Table 3:** Dietary intake, energy correlation (kcal/day) and serum levels of adiponectin, PAI-1, ICAM-1 e VCAM-1

**Variables**	**Association with dietary intake**	**Energy correlation**	
	**p-value**	**r**_**s**_	**p-value**
Adiponectin	0.9	0.8	0.31
PAI-1	0.2	−0.01	0.82
ICAM-1	0.7	0.01	0.99
VCAM-1	0.7	−0.05	0.95

### Correlation between lipid profile, blood glucose and levels of serum adipokines and adhesion molecules

Analysis of total cholesterol, LDL, HDL, triglyceride and glucose and their relationship to serum levels of adiponectin, PAI-1, ICAM-1 and VCAM-1 showed a linear correlation between serum levels of PAI-1 and triglycerides (r^2^=0.07, β = 0.083; p=0.001) (Table 4). This means that for every 10ng/ml increase of triglycerides, there was an increase of 0.8ng/ml in the PAI-1 serum level,

**Table 4:** Correlation of serum levels of adipokines, cell adhesion molecules and metabolic variables

**Variables**	**Adiponectin**		**PAI-1**		**ICAM-1**		**VCAM-1**	
	**r**_**s**_	**p-value**	**r**_**s**_	**p-value**	**r**_**s**_	**p-value**	**r**_**s**_	**p-value**
Total cholesterol	−0.02	0.79	0.05	0.47	−0.05	0.51	−0.1	0.2
HDL cholesterol	0.05	0.48	0.07	0.37	−0.05	0.51	−0.02	0.8
LDL cholesterol	−0.03	0.7	−0.05	−0.5	−0.01	0.21	−0.04	0.57
Triglycerides	−0.01	0.8	0.26	0.001	0.05	0.5	−0.007	0.9
Fasting glucose	0.07	0.34	0.05	0.48	0.01	0.87	0.1	0.19

### Correlation between BMI and serum levels of adipokines and VCAM-1

There was an inverse correlation between VCAM-1 serum levels and BMI (r^2^=−0.22; β=−1.77; p= 0.007). (Table 5). For every 1 kg/m2 increase in BMI, there was a reduction of 1.7ng/ml in the VCAM-1 serum level.

**Table 5:** Association of BMI and WHR with serum levels of adiponectin, PAI-1, ICAM-1 and VCAM-1

**Variables**	**Association with BMI**		**Association with WHR**	
	**r**_**s**_	**p-value**	**r**_**s**_	**p-value**
Adiponectin	0.04	0.58	−0.04	0.62
PAI-1	0.08	0.30	0.16	0.04
ICAM-1	0.05	0.52	0.05	0.49
VCAM-1	−0.22	0.007	−0.02	0.76

### Correlation between waist-to-hip ratio (WHR) and serum levels of adipokines and VCAM-1

There was a linear correlation between PAI-1 serum levels and WHR (r^2^=0.16; β = 108.07; p= 0.04) (Table 5). For an increase of 0.1 units of WHR there was an increase of 10.8ng/ml of PAI-1 levels.

## Discussion

As part of a cohort study of 10,000 woman for the prevention of breast cancer in Porto Alegre, Southern Brazil, the present study has found alarming results, with 39.3% of obesity and 37.2% of overweight among our study population, which was recruited consecutively from the main cohort. In addition, we encountered a significant percentage of women with type 2 diabetes mellitus, hypertension, dyslipidemia and high WHR. One possible explanation for the high prevalence of overweight in our sample may be the low level of education, as 60% of women had completed just elementary education [21]. The relationship between education and obesity has been described in other studies, as the one carried out with the Danish population, which concluded that education was consistently associated with BMI and obesity [22]. Another explanation for the high prevalence of overweight and obesity could be the lower rate of physical activity, since only 35% of the participants were performing exercises on a regular basis.

We found a positive correlation between serum levels of PAI-1 and serum triglycerides. PAI-1 levels correlate significantly with a variety of adiposity measures (BMI, waist circumference, WHR, total fat, visceral adipose tissue), and also with markers of the metabolic syndrome (inflammatory markers, insulin, glucose, triglycerides and high density lipoprotein) [23]. The activity of PAI-1 is associated with insulin resistance, regardless of serum triglycerides and other potential confounding factors [24-27]. Thus, our findings reinforce the relationship of this adipokine with obesity, especially with abdominal obesity [26-29].

There was also a positive correlation between serum levels of PAI-1 and WHR. It was demonstrated that the increase of visceral adipose tissue (VAT) is directly associated with levels of PAI-1, even after adjusting for BMI [30]. Excess of adipose tissue increases the production of PAI-1, leading to an impairment of the fibrinolytic system [30]. PAI-1 concentration varies according to race, ethnicity and gender, although differences in body composition and in the distribution of adipose tissue may be responsible for much of this variability [31].

We showed an inverse correlation between BMI and serum levels of VCAM-1. Although obesity is associated with an increase rather than a decrease in cell adhesion molecules expression, including VCAM-1 [32], the mechanisms that explain the pathophysiological changes which make obesity a risk factor for atherosclerotic diseases, and therefore increase the adhesion molecules, are not yet fully understood. The results of the studies are in fact controversial [33-35].

A study found that elevated levels of VCAM-1 in non-obese patients with polycystic ovary syndrome was associated with insulin resistance, independent of the BMI [36]. We did not measure insulin levels and neither evaluated homeostasis model assessment of insulin resistance index (HOMA IR), which could be helpful in establishing the presence of metabolic syndrome, what could explain variations in VCAM-1 levels.

Adhesion molecules are associated to breast cancer and was shown to be reduced after breast cancer therapy [17], leading to the point that obesity and metabolic syndrome-type characteristics may possibly increase the likelihood of cancer risk. Therefore, there may be significant differences in ICAM and VCAM in patients with and without breast cancer (regardless of obesity and metabolic syndrome) and this should be investigated in future studies.

Since most women in this study were overweight and had increased WHR we would expect to find a correlation of BMI and WHR with ICAM-1. However, this correlation was not found. The serum levels of ICAM-1 are positively correlated with obesity, in particular, to visceral adipose tissue [37]. On the other hand, it was demonstrated that serum levels of ICAM-1 decreases after weight reduction [38]. Since no data was collected on weight modification, we could not evaluate this association.

Contrary to our expectations, there was no correlation between serum levels of adiponectin and the variables associated to overweight and obesity. According to the literature, serum levels of adiponectin are negatively correlated with BMI in male and female individuals as well as with visceral fat [39]. Patients with type 2 diabetes mellitus, hypertension^,^ and breast cancer, for example, also show reduced peripheral levels of adiponectins [40-42]. Biological variations of adiponectin serum concentrations seem to be associated with differences in gender, age and race [43]. Women have 35% higher plasma adiponectin than male individuals [44]. A possible explanation for this profile is that androgens may reduce the concentrations of this biomarker in men [43,44].

It seems that the ratio adiponectin:leptin may be more important than the measure of the absolute level of these biomarkers. Recent studies have shown that the adiponectin:leptin ratio is reduced in women with breast cancer, and that BMI presents a negative and a positive correlation, respectively, with serum levels of adiponectin and leptin [45,46].

## Conclusion

In conclusion, we found a higher prevalence than expected of overweight and obesity in women undergoing mammographic screening in Southern Brazil. PAI-1 levels were correlated to lipid and anthropometric profile and VCAM-1 was inversely related to BMI. Additional studies are necessary to determine if adipokines and adhesion molecules are indeed associated with an increased risk for diseases like type 2 diabetes mellitus, hypertension and breast cancer.

## Competing interests

The authors declare that they have no competing interests.

## Authors’ contributions

MSG and MC conceived of the study and participated in its design and coordination. CIS, BE, GHC, PT and DDR participated in the design of the study. ECN carried out the assays. CIS, BE, GHC, JG and PT collected patient data. LRM performed the statistical analysis. CIS, DDR, PAP, MC, EHM and MSG drafted the manuscript. All authors read and approved the final manuscript.
